# Detection of carbapenemase producing *Acinetobacter baumannii* ST19 from Georgia and Ukraine carrying *bla*_OXA-23_, *bla*_OXA-72_, and/or *bla*_NDM-5_, December 2019 to June 2023

**DOI:** 10.2807/1560-7917.ES.2024.29.24.2400259

**Published:** 2024-06-13

**Authors:** Ting L Luo, Melissa J Martin, Valentyn Kovalchuk, Viacheslav Kondratiuk, Nino Trapaidze, Magda Metreveli, Christine E Hulseberg, Henry D Dao, Yoon I Kwak, Rosslyn Maybank, Thomas A Musich, Matthew R Scherer, Jason W Bennett, Patrick T Mc Gann, Francois Lebreton

**Affiliations:** 1 Multidrug-Resistant Organism Repository and Surveillance Network (MRSN), Walter Reed Army Institute of Research, Silver Spring, Maryland, United States; 2 Department of Microbiology, National Pirogov Memorial Medical University, Vinnytsia, Ukraine; 3 Department of Emergency and Military Medicine, National Pirogov Memorial Medical University, Vinnytsia, Ukraine; 4 US Army Medical Research Directorate - Europe & Middle East, Tbilisi, Georgia; 5 Landstuhl Regional Medical Center, Landstuhl, Germany

**Keywords:** *Acinetobacter baumannii*, *A. baumannii*, surveillance, carbapenem resistance, NDM, nosocomial outbreak, microbial genomics, antimicrobial resistance, molecular epidemiology

## Abstract

In 2003−2023, amid 5,436 *Acinetobacter baumannii* isolates collected globally through the Multidrug-Resistant Organism Repository and Surveillance Network, 97 were ST19^PAS^, 34 of which carbapenem-resistant. Strains (n = 32) sampled after 2019 harboured either *bla*_OXA-23_, *bla*_OXA-72_, and/or *bla*_NDM-5_. Phylogenetic analysis of the 97 isolates and 11 publicly available ST19 genomes revealed three sub-lineages of carbapenemase-producing isolates from mainly Ukraine and Georgia, including an epidemic clone carrying all three carbapenemase genes. Infection control and global surveillance of carbapenem-resistant *A. baumannii* remain important.

Between 2003 and 2023, 97 multidrug resistant (MDR) *Acinetobacter baumannii* isolates of sequence type (ST)19 were identified through the Multidrug-Resistant Organism Repository and Surveillance Network (MRSN) at the Walter Reed Army Institute of Research (WRAIR) in the United States (US) [1,2]. We here describe these ST19 MDR isolates, conduct phylogenomic analyses together with 11 non-redundant ST19 genomes available in GenBank, and raise awareness on the increasing number of carbapenemase-producing ST19 detected in Georgia and Ukraine between 2019 and 2023.

## *Acinetobacter baumannii* ST19 collection and characteristics

From 5,436 *A. baumannii* genomes of MDR strains (as defined by Magiorakos et al. [3]) in the MRSN repository, 97 *A. baumannii* genomes of ST19 were identified. These were derived from isolates cultured from 97 patients between 2003 and 2023. The isolates originated from three distinct sources: (i) wounded US service members evacuated from Iraq and/or Afghanistan to military hospitals in Germany and/or US, between 2003 and 2009, (ii) patients from six hospitals in Georgia between 2011 and 2022 and, (iii) patients hospitalised in Ukraine between 2014 and 2023; more details can be found in the Supplementary Table S1.

Antibiotic susceptibility tests were performed in the MRSN College of American Pathologists accredited clinical laboratory, as previously described [4], and interpreted using the Clinical and Laboratory Standards Institute 2023 guidelines [5]. They revealed that just two of the 63 isolates (3%) collected between 2003 and 2018 were each non-susceptible to both imipenem and meropenem, while 32 of the 34 isolates collected since 2019 were non-susceptible to these two carbapenems, as detailed in Supplementary Table S1.

**Figure 1:**
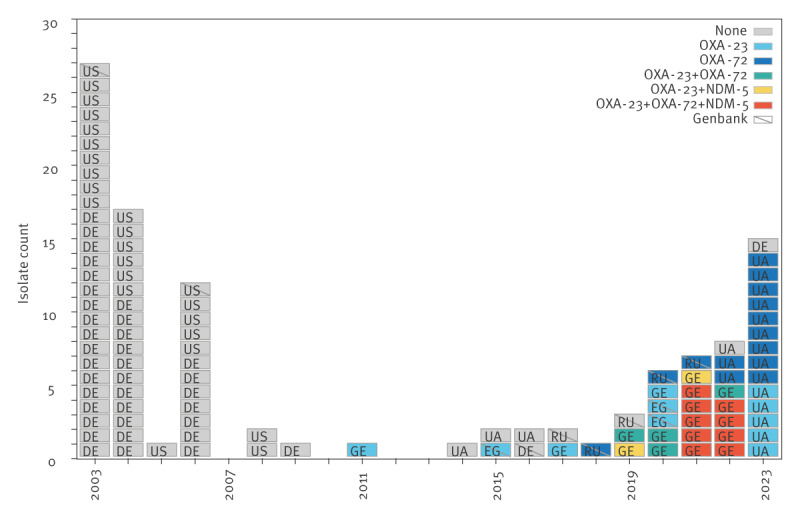
Distribution of *Acinetobacter baumannii* ST19 isolates recovered from several countries^a^, 2003−2023 (n = 108 isolates)^b^

DE: Germany; EG: Egypt; GE: Georgia; NDM: New Dehli metallo-β-lactamase; OXA: oxallicinase; RU: Russia; ST: sequence type; UA: Ukraine; US: United States.

^a^ The countries include DE, EG, GE, RU, UA and the US.

^b^ Data were retrieved from the Multidrug-Resistant Organism Repository and Surveillance Network for 97 isolates and from GenBank for the remaining 11 strains.

Notably, genomic analysis demonstrated that all non-susceptible isolates carried an acquired carbapenemase. The two isolates collected before 2019 originated from Georgia and harboured *bla*_OXA-23_ and the 32 collected since, originated from both Georgia and Ukraine and carried distinct resistance genes: *bla*_OXA-23_ only (n = 6), just *bla*_OXA-72_ (n = 11), *bla*_OXA-23_/*bla*_OXA-72_ (n = 4), *bla*_OXA-23_/*bla*_NDM-5_ (n = 2) and *bla*_OXA-23_/*bla*_OXA-72_/*bla*_NDM-5_ (n = 9) (Figure 1 and Supplementary Table S1).

Finally, all non-redundant ST19 genomes available on GenBank (n = 11) were analysed: only two of six collected before 2019 carried a carbapenemase gene (one from Egypt with *bla*_OXA-23_ and one from Russia with *bla*_OXA-72_) while four of five isolates collected since 2019 carried *bla*_OXA-23_ (n = 2 from Egypt) or *bla*_OXA-72_ (n = 2 from Russia) (Figure 1).

## Three sub-lineages of carbapenemase-producing *Acinetobacter baumannii* ST19 from Georgia and Ukraine

**Figure 2:**
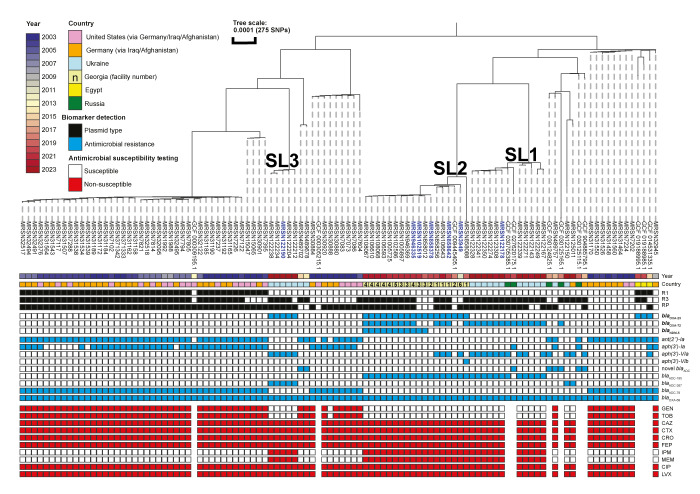
Core genome single nucleotide polymorphism (SNP)-based phylogenetic tree of ST19 *Acinetobacter baumannii* strains recovered in several countries^a^, 2003−2023 (n = 108 strains)

CAZ: ceftazidime; CIP: ciprofloxacin; CRO: ceftriaxone; CTX: cefotaxime; FEP: cefepime; GEN: gentamicin; IPM: imipenem; LVX: levofloxacin; MEM: meropenem; NDM: New Dehli metallo-β-lactamase; OXA: oxallicinase; ST: sequence type; TOB: tobramycin.

^a^ The countries are Egypt, Georgia, Germany (i.e. wounded United States service members evacuated from Iraq and/or Afghanistan), Russia, Ukraine and the United States (i.e. wounded United States service members evacuated from Iraq and/or Afghanistan).

Core genome single nucleotide polymorphism (SNP)-based phylogenetic analysis of all ST19 isolates (n = 108) revealed that, besides three monophyletic isolates from Egypt which harboured *bla*_OXA-23_ [6], carbapenemase-producing isolates grouped into three sub-lineages (SL) (Figure 2).

SL1 comprised 13 isolates and SNP distances in this cluster ranged from 56 to 363. The isolates originated from Ukraine (n = 11, between 2022 and 2023) and Russia (n = 2, from 2020 and 2021) and all bore *bla*_OXA-72_.

SL2 consisted of 17 isolates carrying *bla*_OXA-23_, separated by 40 to 539 SNPs, recovered from six hospitals in Georgia. Two isolates from before 2019 only carried *bla*_OXA-23_, while the remaining 15, collected from that year onwards, incrementally acquired *bla*_OXA-72_ (n = 12) and *bla*_NDM-5_ (n = 11). This culminated with nine highly genetically related isolates (8–14 SNPs), collected from patients in three Georgian hospitals in 2021–2022, carrying all three carbapenemases (Figure 2).

Finally, SL3 represented five highly genetically related isolates (7–42 SNPs) recovered from patients in Ukraine in 2023 and all carrying *bla*_OXA-23_.

## Genetic context of acquired carbapenemases in *Acinetobacter baumannii* ST19

Long-read sequencing was performed on seven representative isolates from SL1, -2 and -3 (Table), as previously described [7]. Based on *rep* typing [8], circularised plasmids belonged to two groups, R3 and RP, except for three cryptic plasmids that carried no resistance genes (Table).

**Table:** Identification of plasmids and location of carbapenemase genes in representative ST19 *Acinetobacter baumannii* isolates with complete genomes, 2011−2023 (n = 7 isolates)

Isolate ID	SL	Plasmids types^a^			Carbapenemases^b^		
		RP (kb)	R3 (kb)	Others (kb)	*bla*_OXA-23_	*bla*_OXA-72_	*bla*_NDM-5_
122178	1	70.5	8.5	NA	NA	R3	NA
3949	2	71.1	NA	NA	Chr (Tn*2008*)	NA	NA
858378	2	NA	NA	NA	Chr (Tn*2008*)	NA	NA
858642	2	73.0	19.6	9.2	Chr (Tn*2008*)	R3	NA
946335	2	69.6	18.1	NA	Chr (Tn*2008*)	R3	Chr (ΔTn*125*)
102519	2	NA	NA	NA	Chr (Tn*2008*)	NA	Chr (ΔTn*125*)
122190	3	98.4	12.7	2.3 and 3.6	Chr (AbaR4), RP (AbaR4)	NA	NA

ID: identification number; NA: not applicable; SL: sub-lineage; ST: sequence type.

^a^ When present, the plasmid size is indicated for RP- and R3-types as well as for cryptic plasmids (others).

^b^ Carbapenemase gene inferred location on the chromosome (Chr) or plasmids (RP- and R3-types). When applicable, the carriage by transposons (Tn*2008* or truncated ΔTn*125*) or resistance island (AbaR4) is indicated.

**Figure 3:**
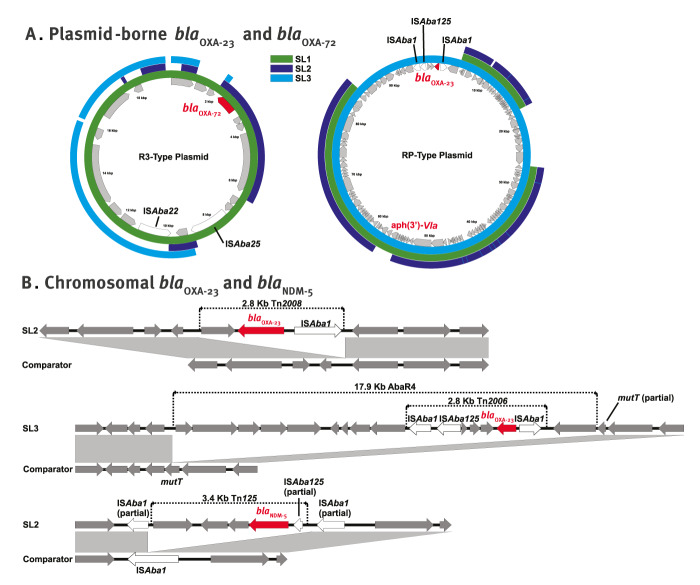
Genetic context of carbapenemase genes present in the three sub-lineages of *Acinetobacter baumannii* ST19

NDM: New Dehli metallo-β-lactamase; OXA: oxallicinase; SL: sub-lineage; ST: sequence type.

For completeness, plasmid *rep* types were also identified for all the remaining ST19 isolates using Illumina short reads and mapped onto the phylogeny (Figure 2). The three carbapenemase genes, *bla*_OXA-23,_ *bla*_OXA-72_ and *bla*_NDM-5_ found in the three SLs appear to be independent acquisitions. For SL1, *bla*_OXA-72_ was plasmid-bound (R3-type) and no significant homologies to composite transposons in the TnCentral database [9] were identified (Figure 3A). Concerning SL2, *bla*_OXA-23_ was chromosomal and carried by transposon Tn*2008* [10] (Figure 3B). When present in SL2 isolates, *bla*_NDM-5_ was also chromosomal and carried by a 3.4 kb fragment of a truncated Tn*125*, inserted within IS*Aba1*. In contrast, when present in SL2 isolates, *bla*_OXA-72_ was on a plasmid (R3-type) (Table and Figure 3). Of note, R3 plasmids from SL1 and SL2 isolates varied in length from 8.5 to 19.6 kb and an alignment confirmed that each lineage had distinct plasmid backbones despite an identical 7.0 kb insert harbouring *bla*_OXA-72_ (Figure 3A)_._ Finally, for the representative SL3 isolate, two identical copies of *bla*_OXA-23_ carried on resistance island AbaR4 were identified: one flanked by a 5 bp (5′-AAGGG-3′) target site duplication (TSD) within a RP-type plasmid and the second inserted within the chromosome (Figure 3). Interestingly, in this SL, and unlike a previous report [11], AbaR4 did not replace the ancestral AbaR3 island which remained intact within gene *comM*. Instead, AbaR4 was located ca 30 kb downstream, flanked by a 5′-AACTT-3′ TSD within chromosomal gene *mutT*.

## Discussion

Multidrug-resistant *A. baumannii* has emerged as a leading cause of nosocomial infections worldwide [12]. Of particular concern is the high rate of resistance to carbapenem antibiotics (> 50% in 21 European countries in 2020-2022 [13]), that makes treatment difficult [14]. Carbapenem resistance in *A. baumannii* is generally attributed to either mutation and overexpression of the intrinsic OXA-51-like carbapenemase or the acquisition of more potent carbapenemases (most often OXA-23 and OXA-24/40-like enzymes) via horizontal gene transfer [15].

The acquisition of carbapenemases, via horizontal gene transfer, is well characterised in ST1 and ST2, the preeminent lineages identified by multilocus sequence typing within global clones, GC1 and GC2, respectively [12,16]. Nevertheless, GC complexes also include other lineages. One of these is ST19, a single locus variant of the ST1/GC1 clone, that has scarcely been reported globally [6,17-20]. At the time of the current study, we could find only 11 non-redundant genomes for *A. baumann*ii ST19 in the GenBank database and, to the best of our knowledge, just five published reports of studies relating to this ST [6,17-20]. The first of these articles, published in 2015, described the detection of four ST19 isolates from hospital surfaces in Algeria, carrying *bla*_NDM-5_ [17]. The second concerned a blood culture ST19 isolate in 2017 from a patient in Georgia, which harboured *bla*_OXA-23_ [20]. The third in 2019, described ST19 isolates from an intensive care unit (ICU) in Italy [18]. The fourth concerned three isolates in 2017−2019 from a neurological ICU in Moscow, Russia, all harbouring *bla*_OXA-72_ (an OXA-24/40 variant) [19] and the fifth was an article on three ST19 isolates from 2020 in Egypt and carrying *bla*_OXA-23_ on a chromosomally inserted Tn*2006* transposon [6].

Amid 5,436 *A. baumannii* strains from 2003−2023 available in the MRSN repository, we found 97 of ST19. Among these ST19 strains, the proportions with carbapenem resistance appeared to be less (2/63) before 2019 than after (32/34) and, similarly, among 11 non-redundant ST19 genome sequences in GenBank, two of six from pre-2019 carried a carbapenemase gene while this was four of five post-2019*.* This could suggest that carbapenem-resistant *A. baumannii* ST19 are emerging or alternatively that these have previously been under-reported. Routine sampling accompanied by field epidemiology would be needed to fully understand their prevalence. Core genome SNP-based phylogenetic analysis of all 97 MRSN and 11 GenBank ST19 sequences revealed that, besides three monophyletic isolates from Egypt, carbapenemase-producing isolates grouped into three SLs, including SL1, -2 and 3. The MRSN ST19 isolates that had been collected from 2019 onwards and that were non-susceptible to carbapenems harboured either *bla*_OXA-23_, *bla*_OXA-72_, and/or *bla*_NDM-5_ genes and were from Georgia and Ukraine. These isolates were found among the three SLs characterised in the current study.

Some isolates in SL2 accrued up to three carbapenemases, including *bla*_NDM-5_ (which is relatively rare in this species) [21,22]. Thus, like other GC1 lineages [12], ST19 appears prone to acquiring carbapenem resistance genes, a phenomenon also illustrated by the recurring introductions of *bla*_OXA-23_. Indeed, including isolates from Egypt, *bla*_OXA-23_ was acquired on at least three occasions in ST19 via the transfer of: (i) transposon Tn*2008* (SL2 in Georgia), (ii) Tn2006 (Egypt [6]) or (iii) resistance island AbaR4 (SL3 in Ukraine).

A few limitations to this study can be noted. First, while the MRSN collected MDR *A. baumannii* from more than 20 countries on five continents [4], the presence of ST19 in other regions of the world remains to be investigated. Further, the volume and geographical origin of isolates sampled by the MRSN is changing through time. Hence, representative, and regular sampling and typing would be needed to better understand the prevalence of carbapenemase-producing ST19 *A. baumannii* globally. Finally, clonal isolates were not epidemiologically investigated. SL3, for instance, represents five highly genetically related isolates (7–42 SNPs) carrying *bla*_OXA-23_ recovered from patients hospitalised in Ukraine in the same year (2023). It cannot be ruled out that isolates in SL3 represent an outbreak related to a single hospital. This could have inflated the numbers of ST19 isolates bearing carbapenemase genes, in particular for those collected after 2019.

## Conclusion

While it is not possible to assess if incidence of carbapenemase-producing ST19 *A. baumannii* has recently increased in certain parts of the world, or if such strains have so far been under-detected, this report sheds more light on ST19, a ST barely described in the literature, yet circulating in several countries. The finding of multiple SLs of carbapenemase-producing ST19 *A. baumannii* from Georgia and Ukraine is concerning, as this might be exacerbated by the Russian invasion of Ukraine, which is causing disruption in infection prevention and control and movements of trauma patients. Learning from past protracted *A. baumannii* outbreaks occurring in US hospitals during military operations in Iraq and Afghanistan [23], it is imperative that programmes to detect carbapenemase-producing *A. baumannii* in facilities treating war wounded people be implemented and/or reinforced. Finally, this report further emphasises the key role of global epidemiological and genomic surveillance programmes to address antimicrobial resistance.

## Supplementary Data

44000 Supplement

## References

[1] Lesho E Craft D Kirkup BC Jr Waterman P Summers A Vahey MT Surveillance, characterisation, and preservation of multidrug-resistant bacteria. Lancet Infect Dis. 2011;11(1):8-10. 10.1016/S1473-3099(10)70261-9 21183141

[2] Waterman P Kwak Y Clifford R Julius M Onmus-Leone F Tsurgeon C A multidrug-resistance surveillance network: 1 year on. Lancet Infect Dis. 2012;12(8):587-8. 10.1016/S1473-3099(12)70149-4 22835896

[3] Magiorakos AP Srinivasan A Carey RB Carmeli Y Falagas ME Giske CG Multidrug-resistant, extensively drug-resistant and pandrug-resistant bacteria: an international expert proposal for interim standard definitions for acquired resistance. Clin Microbiol Infect. 2012;18(3):268-81. 10.1111/j.1469-0691.2011.03570.x 21793988

[4] Galac MR Snesrud E Lebreton F Stam J Julius M Ong AC A diverse panel of clinical Acinetobacter baumannii for research and development. Antimicrob Agents Chemother. 2020;64(10):e00840-20. 10.1128/AAC.00840-20 32718956 PMC7508605

[5] Clinical and Laboratory Standards Institute (CLSI). Performance Standards for Antimicrobial Susceptibility Testing. 33rd ed. CLSI Supplement M100. CLSI; 2023. Available from: https://clsi.org/media/tc4b1paf/m10033_samplepages-1.pdf

[6] Hamed SM Hussein AFA Al-Agamy MH Radwan HH Zafer MM . Genetic configuration of genomic resistance islands in Acinetobacter baumannii clinical isolates from Egypt. Front Microbiol. 2022;13:878912. 10.3389/fmicb.2022.878912 35935207 PMC9353178

[7] Russo TA Alvarado CL Davies CJ Drayer ZJ Carlino-MacDonald U Hutson A Differentiation of hypervirulent and classical Klebsiella pneumoniae with acquired drug resistance. MBio. 2024;15(2):e0286723. 10.1128/mbio.02867-23 38231533 PMC10865842

[8] Lam MMC Koong J Holt KE Hall RM Hamidian M . Detection and typing of plasmids in Acinetobacter baumannii using *rep* genes encoding replication initiation proteins. Microbiol Spectr. 2023;11(1):e0247822. 10.1128/spectrum.02478-22 36472426 PMC9927589

[9] Ross K Varani AM Snesrud E Huang H Alvarenga DO Zhang J TnCentral: a Prokaryotic transposable element database and web portal for transposon analysis. MBio. 2021;12(5):e0206021. 10.1128/mBio.02060-21 34517763 PMC8546635

[10] Nigro S Hall RM . Distribution of the blaOXA-23-containing transposons Tn2006 and Tn2008 in Australian carbapenem-resistant Acinetobacter baumannii isolates. J Antimicrob Chemother. 2015;70(8):2409-11. 10.1093/jac/dkv102 25881617

[11] Hamidian M Hall RM . AbaR4 replaces AbaR3 in a carbapenem-resistant Acinetobacter baumannii isolate belonging to global clone 1 from an Australian hospital. J Antimicrob Chemother. 2011;66(11):2484-91. 10.1093/jac/dkr356 21873287

[12] Hamidian M Nigro SJ . Emergence, molecular mechanisms and global spread of carbapenem-resistant Acinetobacter baumannii. Microb Genom. 2019;5(10):e000306. 10.1099/mgen.0.000306 31599224 PMC6861865

[13] European Centre for Disease Prevention and Control (ECDC). Antimicrobial resistance surveillance in Europe 2022-2020 data. Stockholm: ECDC; 2022. Available from: https://www.ecdc.europa.eu/en/publications-data/antimicrobial-resistance-surveillance-europe-2022-2020-data

[14] Tamma PD, Aitken SL, Bonomo RA, Mathers AJ, van Duin D, Clancy CJ. Infectious Diseases Society of America 2023 guidance on the treatment of antimicrobial resistant gram-negative infections. Clin Infect Dis. 2023;ciad428. Epub ahead of print. 10.1093/cid/ciad428 37463564

[15] Poirel L Nordmann P . Carbapenem resistance in Acinetobacter baumannii: mechanisms and epidemiology. Clin Microbiol Infect. 2006;12(9):826-36. 10.1111/j.1469-0691.2006.01456.x 16882287

[16] Diancourt L Passet V Nemec A Dijkshoorn L Brisse S . The population structure of Acinetobacter baumannii: expanding multiresistant clones from an ancestral susceptible genetic pool. PLoS One. 2010;5(4):e10034. 10.1371/journal.pone.0010034 20383326 PMC2850921

[17] Zenati K Touati A Bakour S Sahli F Rolain JM . Characterization of NDM-1- and OXA-23-producing Acinetobacter baumannii isolates from inanimate surfaces in a hospital environment in Algeria. J Hosp Infect. 2016;92(1):19-26. 10.1016/j.jhin.2015.09.020 26615460

[18] Lorenzin G Scaltriti E Gargiulo F Caccuri F Piccinelli G Gurrieri F Extensively drug-resistant Acinetobacter baumannii isolated from intensive care units in northern Italy: a genomic approach to characterize new sequence types. Future Microbiol. 2019;14(15):1281-92. 10.2217/fmb-2019-0083 31638422

[19] Fursova NK Fursov MV Astashkin EI Fursova AD Novikova TS Kislichkina AA Multidrug-resistant and extensively drug-resistant Acinetobacter baumannii causing nosocomial meningitis in the neurological intensive care unit. Microorganisms. 2023;11(8):2020. 10.3390/microorganisms11082020 37630581 PMC10458171

[20] Farlow J Nozadze M Mitaishvili N Kotorashvili A Kotoria N Arobelidze K Comparative genomic analysis of four multidrug-resistant isolates of Acinetobacter baumannii from Georgia. J Glob Antimicrob Resist. 2020;21:363-8. 10.1016/j.jgar.2019.11.002 31730823

[21] Poirel L Bonnin RA Nordmann P . Genetic basis of antibiotic resistance in pathogenic Acinetobacter species. IUBMB Life. 2011;63(12):1061-7. 10.1002/iub.532 21990280

[22] Kitti T Manrueang S Leungtongkam U Khongfak S Thummeepak R Wannalerdsakun S Genomic relatedness and dissemination of *bla* _NDM-5_ among Acinetobacter baumannii isolated from hospital environments and clinical specimens in Thailand. PeerJ. 2023;11:e14831. 10.7717/peerj.14831 36778153 PMC9912941

[23] Luo TL Harmer CJ Lebreton F Stam J Bennett JW Hall RM Identification of an outbreak cluster of extensively antibiotic-resistant GC1 Acinetobacter baumannii isolates in U.S. military treatment facilities. Microbiol Spectr. 2023;11(3):e0046223. 10.1128/spectrum.00462-23 37140387 PMC10269654

